# Population Pharmacokinetic and Concentration-QTc Analysis of Delamanid in Pediatric Participants with Multidrug-Resistant Tuberculosis

**DOI:** 10.1128/AAC.01608-21

**Published:** 2022-02-15

**Authors:** Tomohiro Sasaki, Elin M. Svensson, Xiaofeng Wang, Yanlin Wang, Jeffrey Hafkin, Mats O. Karlsson, Suresh Mallikaarjun

**Affiliations:** a Otsuka Pharmaceutical Co., Ltd., Osaka, Japan; b Department of Pharmacy, Uppsala University, Uppsala, Sweden; c Department of Pharmacy, Radboud University Medical Center, Nijmegen, The Netherlands; d Otsuka Pharmaceutical Development & Commercialization, Inc., Rockville, Maryland, USA

**Keywords:** *Mycobacterium tuberculosis*, PK/QTc, delamanid, pediatric drug therapy, population PK

## Abstract

A population pharmacokinetic analysis of delamanid and its major metabolite DM-6705 was conducted to characterize the pharmacokinetics of delamanid and DM-6705 in pediatric participants with multidrug-resistant tuberculosis (MDR-TB). Data from participants between the ages of 0.67 and 17 years, enrolled in 2 clinical trials, were utilized for the analysis. The final data set contained 634 delamanid and 706 DM-6705 valid plasma concentrations from 37 children. A transit model with three compartments best described the absorption of delamanid. Two-compartment models for each component with linear elimination were selected to characterize the dispositions of delamanid and DM-6705, respectively. The covariates included in the model were body weight on the apparent volume of distribution and apparent clearance (for both delamanid and DM-6705); formulation (dispersible versus film-coated tablet) on the mean absorption time; age, formulation, and dose on the bioavailability of delamanid; and age on the fraction of delamanid metabolized to DM-6705. Based on the simulations, doses for participants within different age/weight groups that result in delamanid exposure comparable to that in adults following the approved adult dose were calculated. By concentration-QTc (QTcB [QT corrected by Bazett’s formula]) analysis, a significant positive correlation was detected with concentrations of DM-6705. However, the model-predicted upper bounds of the 90% confidence intervals of ΔQTc values were <10 ms at the simulated maximum concentration (*C*_max_) of DM-6705 following the administration of the maximum doses simulated. This suggests that the effect on the QT interval following the proposed dosing is unlikely to be clinically meaningful in children with MDR-TB who receive delamanid.

## INTRODUCTION

Tuberculosis (TB), caused by Mycobacterium tuberculosis, remains one of the major causes of death from an infectious disease globally. Based on the 2019 global TB report by the World Health Organization (WHO), an estimated 10.0 million people fell ill with TB in 2018 (1). In that report, 55% of HIV-negative people who died from TB were men (aged ≥15 years), 31% were women, and 14% were children (aged <15 years).

Delamanid (OPC-67683) (Deltyba; Otsuka Pharmaceutical Co., Ltd., Tokyo, Japan) is a bicyclic nitroimidazooxazole compound that inhibits the synthesis of mycolic acids (2), the key components of the lipid-rich cell wall of M. tuberculosis (3). Delamanid has been confirmed to have mycobacterium-specific antibacterial activity *in vitro* and potent antituberculosis activity *in vivo* by oral administration (4). In adults, two randomized, placebo-controlled trials (trial 204 [ClinicalTrials.gov identifier NCT00685360] and trial 213 [ClinicalTrials.gov identifier NCT01424670]) assessed the efficacy and safety of delamanid plus an optimized background regimen (OBR) in the treatment of multidrug-resistant tuberculosis (MDR-TB) (5, 6). Delamanid was approved in adults for the treatment of pulmonary MDR-TB in combination with an OBR in the European Union, Japan, and South Korea in 2014 and has subsequently been approved in several other countries.

The primary safety concern with delamanid is QT prolongation. Evidence suggests that prolongation of the QT interval is correlated with plasma concentrations of delamanid and its major metabolite DM-6705. Using the time-averaged method for assessing the QT interval corrected by Fridericia’s formula (QTcF), delamanid showed a dose-response relationship with an increase in the placebo-corrected QTcF from day 1 to day 56 with a 12-ms mean change at the 100-mg twice-daily (BID) dose level and a 15-ms mean change at the 200-mg twice-daily dose level. Nevertheless, no episodes of QT interval prolongation were accompanied by symptoms (i.e., syncope) or clinically significant arrhythmias (i.e., torsade de pointes) in the clinical development of delamanid.

A pediatric investigational plan (PIP) for delamanid, agreed upon in consultation with the European Medicines Agency (EMA), was launched to ensure the optimal use of delamanid in pediatric MDR-TB participants (7). Per the PIP, 3 clinical trials were conducted: a pharmacokinetic (PK) trial (trial 232 [ClinicalTrials.gov identifier NCT01856634]) in children <18 years of age with MDR-TB who were on therapy with OBR, followed by a 6-month safety and tolerability extension trial (trial 233 [ClinicalTrials.gov identifier NCT01859923]) in the same subject population, and a bioavailability/bioequivalence trial (trial 245) in adults to investigate the comparative bioavailability of the pediatric formulation (5- and 25-mg dispersible tablets) with the delamanid adult formulation (50-mg film-coated tablet). Based on the preliminary results from trials 232 and 233, the WHO has recommended the use of delamanid in children aged 6 years and above with MDR-TB with limited treatment options: 50 mg BID for 6 months in children (aged 6 to 11 years) and 100 mg BID for 6 months in adolescents (aged 12 to 17 years) (8). Furthermore, delamanid has been approved by the EMA for children with MDR-TB weighing at least 30 kg as of 2020 (9), and this was extended to children with MDR-TB weighing at least 10 kg very recently (10).

The purpose of this population PK analysis is to further characterize the PK of delamanid in the pediatric population with data collected in trials 232 and 233 and to calculate doses for pediatric participants across the entire age range, i.e., between 0 and 17 years of age, that will result in systemic exposures of delamanid comparable to that of the approved dose in adults (100 mg BID). Furthermore, in order to evaluate the risk of QT prolongation in the pediatric population, a concentration-QT (cQT) analysis was performed for the baseline-corrected QT interval as a function of delamanid and DM-6705 plasma concentrations using data collected in trials 232 and 233.

## RESULTS

### Data included in population PK analysis.

**FIG 1:**
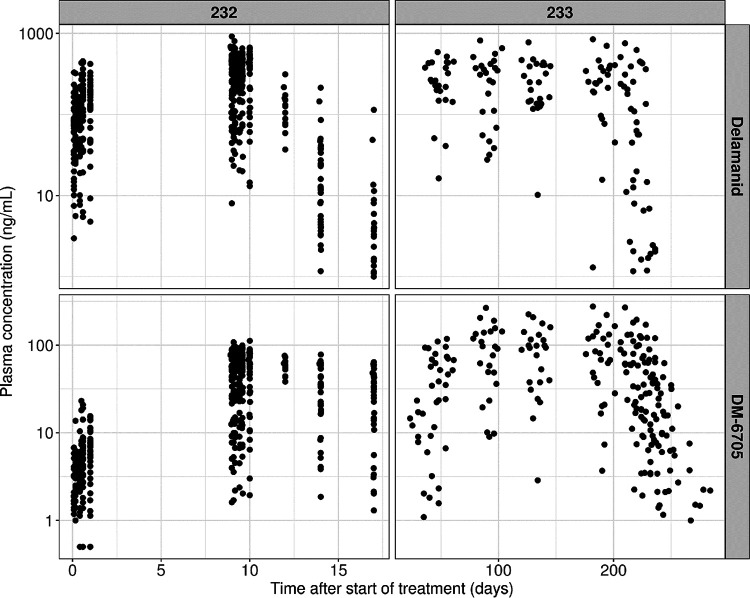
Pharmacokinetic observations stratified by study and analyte-plasma concentration versus time after the first dose.

The final data set contained 634 and 706 valid delamanid and DM-6705 plasma concentrations, respectively, from 37 participants. Figure 1 shows the observed plasma concentrations of delamanid and DM-6705. Summary statistics for covariates included in this analysis from those participants are shown in Table 1.

**TABLE 1:** Summary of covariates included in the population pharmacokinetic analysis

Covariate	Value for group				
	1 (12–17 yrs) (*n* = 7)	2 (6–11 yrs) (*n* = 6)	3 (3–5 yrs) (*n* = 12)	4 (birth–2 yrs) (*n* = 12)	Overall (*n* = 37)
Categorical covariates [no. (%) of participants]					
Sex					
Male	4 (57.1)	2 (33.3)	6 (50)	6 (50)	18 (48.6)
Female	3 (42.9)	4 (66.7)	6 (50)	6 (50)	19 (51.4)
Race					
Black	0 (0.0)	0 (0.0)	2 (16.7)	0 (0.0)	2 (5.41)
Asian	7 (100)	4 (66.7)	8 (66.7)	6 (50)	25 (67.6)
Others	0 (0.0)	2 (33.3)	2 (16.7)	6 (50)	10 (27)
Country					
Philippines	7 (100)	4 (66.7)	8 (66.7)	6 (50)	25 (67.6)
South Africa	0 (0.0)	2 (33.3)	4 (33.3)	6 (50)	12 (32.4)
Dose (mg)					
5	0 (0.0)	0 (0.0)	0 (0.0)	6 (50)	6 (16.2)
10	0 (0.0)	0 (0.0)	0 (0.0)	6 (50)	6 (16.2)
25	0 (0.0)	0 (0.0)	12 (100)	0 (0.0)	12 (32.4)
50	0 (0.0)	6 (100)	0 (0.0)	0 (0.0)	6 (16.2)
100	7 (100)	0 (0.0)	0 (0.0)	0 (0.0)	7 (18.9)
Formulation					
Film-coated tablet	7 (100)	6 (100)	0 (0.0)	0 (0.0)	13 (35.1)
Dispersible tablet	0 (0.0)	0 (0.0)	12 (100)	12 (100)	24 (64.9)

Continuous covariates [mean (SD)]					
Age (yrs)	15.3 (1.62)	9.44 (1.51)	4.3 (0.988)	1.68 (0.591)	6.36 (5.19)
wt (kg)	39 (4.59)	24.9 (6.79)	14.2 (3.2)	9.76 (1.83)	19.2 (11.6)
Body surface area (m^2^)	1.29 (0.0827)	0.917 (0.164)	0.619 (0.0948)	0.461 (0.0632)	0.743 (0.322)
Body mass index (kg/m^2^)	16.6 (2.28)	16.3 (2.4)	14.8 (1.43)	15.9 (2.15)	15.7 (2.04)
Estimated glomerular filtration rate (mL/min/1.73 m^2^)	181 (16.3)	144 (43.4)	159 (46.2)	138 (38.8)	154 (40.9)
Albumin (g/dL)	3.94 (0.613)	4.37 (0.413)	4.42 (0.277)	4.31 (0.348)	4.29 (0.421)
Alanine aminotransferase (U/L)	8.14 (4.14)	8.33 (3.14)	12.4 (4.81)	18.2 (27.9)	12.8 (16.4)
Aspartate aminotransferase (U/L)	22.6 (7.41)	27.5 (5.24)	42.4 (8.72)	51.8 (40.6)	39.3 (26)
Alkaline phosphatase (U/L)	172 (98.4)	240 (99.9)	189 (47.7)	202 (39.2)	198 (67.9)
Total bilirubin (mg/dL)	0.286 (0.09)	0.283 (0.0983)	0.217 (0.0389)	0.241 (0.106)	0.249 (0.086)
Total protein (g/dL)	7.83 (0.528)	7.1 (0.777)	7.69 (0.74)	7.05 (0.598)	7.41 (0.726)

### Population pharmacokinetic modeling of delamanid.

A model was developed previously using the data from trial 232 only. This model included a 2-compartment model with a transit compartment absorption model (number of transit compartments = 1) and linear elimination with interindividual variability (IIV) on the apparent clearance (CL/*F*) and central volume of distribution (*V*_c_/*F*), interoccasional variability (IOV) on bioavailability (*F*_1_) and mean transit time (mean absorption time [MAT]), and a proportional residual error. This model was used as an initial structural model for the current modeling process. Also, based on the previous modeling, the following covariates were included in the model: body weight (BW) on CL/*F*, *V*_c_/*F*, apparent intercompartmental clearance (*Q*/*F*), and peripheral volume of distribution (*V*_p_/*F*); age on *F*_1_ only for ages of <2 years; dose on *F*_1_; and evening administration on MAT.

To describe the absorption phase, 1st-order absorption with lag time and the addition of transit compartments were tested. The model fit was not improved with the 1st-order absorption with lag time model (resulted in an increase of 3.35 points in the Akaike information criterion [AIC]). By adding transit compartments, the model fit was improved, and a number of transit compartments of 3 best described the data (delta AIC = −11.2 versus the model with 1 transit compartment). The addition of IIV on *F*_1_, absorption rate constant (MAT), and *Q* did not improve the model fit. A one-compartment/three-compartment disposition model was tested, but no further improvement in the model was observed. The addition of IIV on *V*_p_/*F* significantly reduced the objective function value (OFV). Also, IIV on *V*_c_/*F* was not statistically significant and was thus removed. Additive and combined (additive and proportional) residual-error models were tested, but no further improvement in the model was observed. In summary, the base model was a two-compartment model with transit compartment absorption (number of transit compartments = 3) and linear elimination with interindividual variability on CL/*F* and *V*_p_/*F*, interoccasional variability on *F*_1_ and MAT, and a proportional residual error.

**FIG 2:**
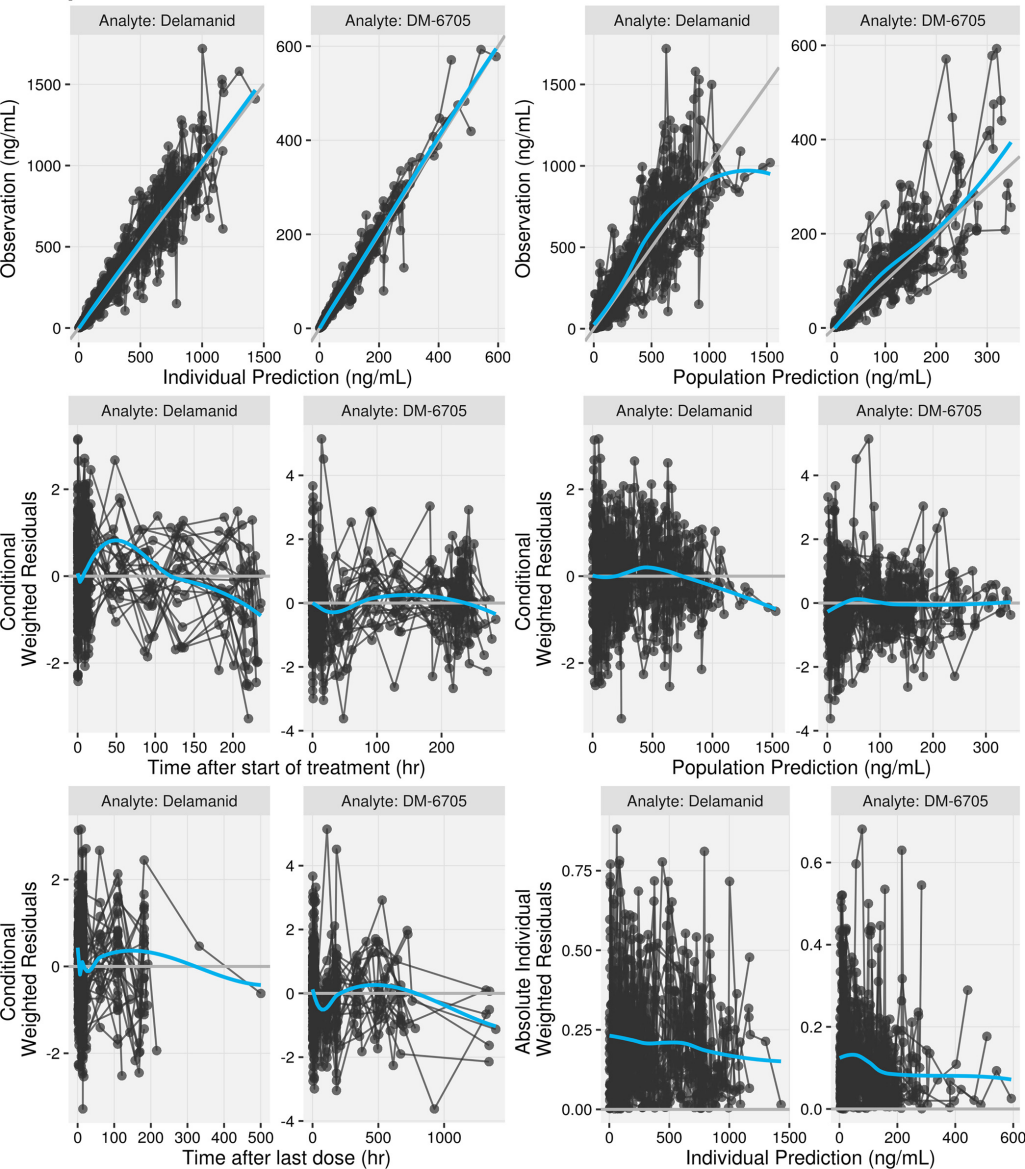
Goodness-of-fit plot of the final model stratified by analyte. Black circles represent individual data. A connected line indicates data from the same individual. Gray lines represent the line of identity (*y* = *x*) or *y* = 0. Blue lines are the smoothing lines of the data.

**FIG 3:**
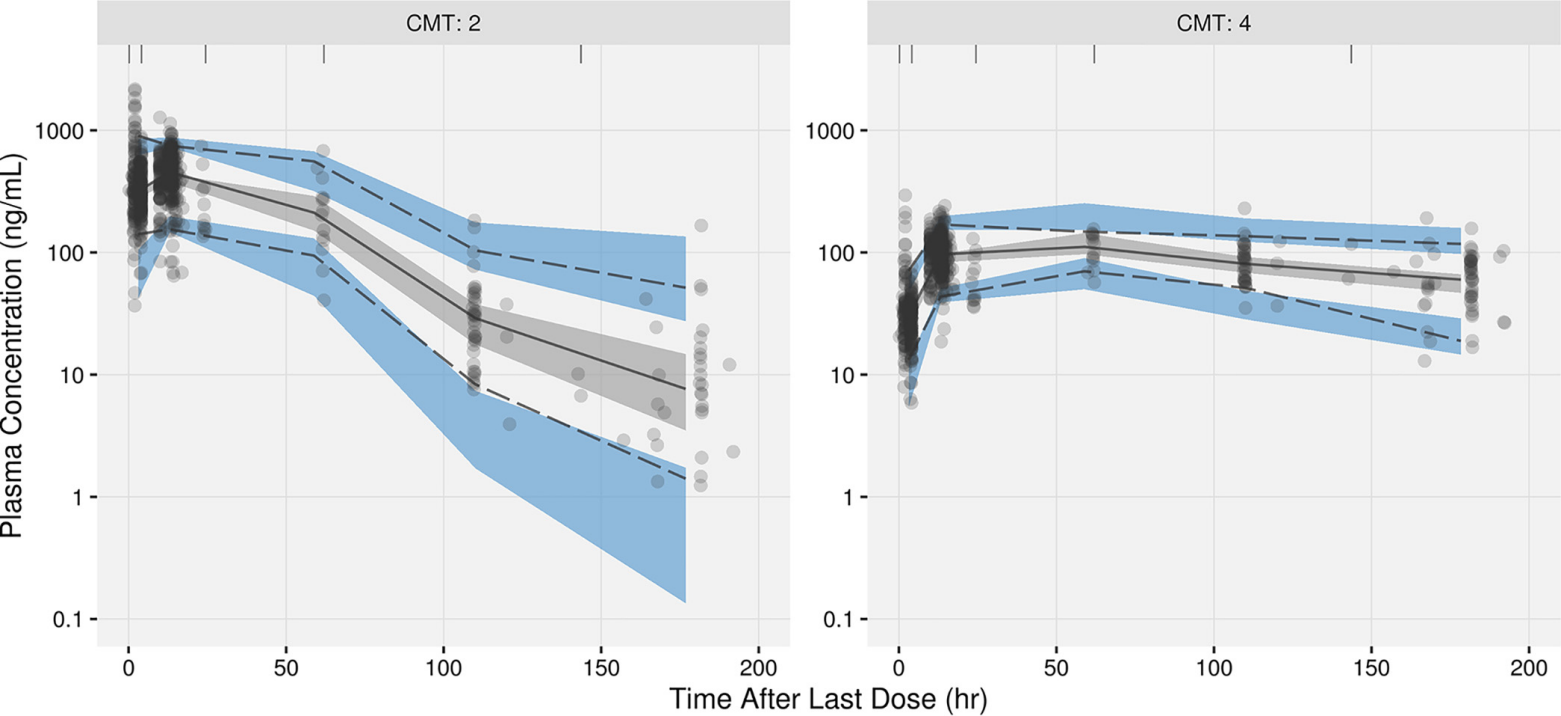
Prediction-corrected visual predictive check plots by analyte. “CMT: 2” represents data for delamanid and “CMT: 4” represents data for DM-6705.

Formulation effects (dispersible tablet versus film-coated tablet) on *F*_1_ and MAT are expected but could not be estimated on the pediatric data alone. To enable simulations accounting for the formulation effect, the estimates from the population PK model including the adult bioequivalence study (trial 245) data were incorporated into the model and fixed. This resulted in an increase of 13.6 points in the OFV. Prespecified covariate effects (see Table S3 in the supplemental material) were evaluated in a stepwise manner. After the forward-addition steps, body mass index (BMI) and total protein were identified as covariates with statistically significant effects on CL/*F*. In addition, albumin and sex were identified as covariates with statistically significant effects on *F*_1_ and *V*_c_/*F*, respectively. The effect of the evening dose on MAT was removed during backward exclusion. All other covariates, including the preincluded ones, were retained after backward exclusion. Based on the clinical relevance and colinearity of BW/BMI and albumin/total protein, BMI and total protein were excluded from the model. Also, the significance of albumin and sex was primarily driven by one or a few individuals with limited clinical importance (Fig. S1). Thus, these two covariates were also excluded from the covariate model. The final covariate model (after these model refinement steps) contained BW on CL/*F*, *V*_c_/*F*, *Q*/*F*, and *V*_p_/*F*; formulation on MAT and *F*_1_; age on *F*_1_ only for ages of <2 years; and dose on *F*_1_. The final parameter estimates are summarized in Table 2. Figure 2 presents goodness-of-fit (GOF) plots for the final population PK model. The prediction-corrected visual predictive checks (pcVPCs) are shown in Fig. 3. The GOF plots and pcVPCs stratified by age group are shown in Fig. S2 and S3, respectively.

**TABLE 2:** Final parameter estimates of the population pharmacokinetic model of delamanid/DM-6705^*c*^

Parameter	Estimate	%RSE
Structural model parameters of delamanid		
CL/*F* (L/h)	17.2	3.36
* V*_c_/*F* (L)	346	8.15
* Q*/*F* (L/h)	62.4	17.6
* V*_p_/*F* (L)	296	13.8
MAT (h)	2.73	7.41

Structural model parameters of DM-6705		
CLM/*F* (L/h)	54.2	6.61
* V*_c_M/*F* (L)	77.0	46.0
* Q*M/*F* (L/h)	425	5.34
* V*_p_M/*F* (L)	13,150	4.27

Covariate effect		
Allometric exponent for wt on CL/*F*, *Q*/*F*, CLM/*F*, and *Q*M/*F*	0.75	Fixed
Allometric exponent for wt on *V*_c_/*F*, *V*_p_/*F*, *V*_c_M/*F*, and *V*_p_M/*F*	1.00	Fixed
Formulation (dispersible tablet) on MAT	0.495	Fixed^*a*^
Formulation (dispersible tablet) on *F*_1_	−0.158	Fixed^*a*^
Age on *F*_1_: linear slope below 2 yrs (1/yrs)	0.201	35.5
Age on FM: linear slope below 6 yrs (1/yrs)	0.0654	17.9
Dose on *F*_1_: increase dose = 50 mg and lower	0.580	Fixed^*b*^

IIV/IOV (CV%)		
IIV on CL/*F*	16.3	32.9
Correlation IIV term between CL/*F* and CLM/*F*	71.0	29.7
IIV on CLM/*F*	33.9	25.5
IIV on *V*_p_/*F*	58.5	34.6
IIV on FM	13.5	39.8
IOV on *F*_1_	26.8	16.2
IOV on MAT	60.7	23.8

RV (CV%)		
Proportional error, delamanid	30.8	7.25
Correlation RV term between delamanid and DM-6705	39.4	14.5
Proportional error, DM-6705	18.1	7.23

a The formulation effect (dispersible tablet versus film-coated tablet) on *F*_1_ and MAT was fixed to the estimates from the population PK model including adult bioequivalence study (trial 245) data.

b Fixed based on the estimates from the adult population PK model using data with a dose range of ∼50 to 400 mg (29).

c CL/*F*, clearance; *V*_c_/*F*, central volume of distribution; *Q*/*F*, intercompartmental clearance; *V*_p_/*F*, peripheral volume of distribution; MAT, mean absorption time; CLM/*F*, clearance of DM-6705; *V*_c_M/*F*, central volume of distribution of DM-6705; *Q*M/*F*, intercompartmental clearance of DM-6705; *V*_p_M/*F*, peripheral volume of distribution of DM-6705; IIV, interindividual variability; IOV, interoccasional variability; CV%, percent coefficient of variation; *F*_1_, bioavailability; FM, fraction metabolized; RV, residual variability; %RSE, percent relative standard error. Standard errors of estimates are obtained by the sampling importance resampling (SIR) approach. Covariate effects for delamanid are modeled as follows: CL/*F* (L/h) = 17.2 × [BW (kg)/33.5]^0.75^, *V*_c_/*F* (L) = 346 × [BW (kg)/33.5]^1.0^, *Q*/*F* (L/h) = 62.4 × [BW (kg)/33.5]^0.75^, *V*_p_/*F* (L) = 296 × [BW (kg)/33.5]^1.0^, MAT = 2.73 × (1 + 0.495) (if the formulation is a dispersible tablet), and *F*_1_ = 1.0{1 − 0.201 × [2 − age (years)]} (if age is >2.0 years) × (1 − 0.158) (if the formulation is a dispersible tablet) × (1 + 0.580) (if the dose is ≤50 mg). Covariate effects for DM-6705 are modeled as follows: CLM/*F* (L/h) = 54.2 × [BW (kg)/33.5]^0.75^, *V*_c_M/*F* (L) = 77.0 × [BW (kg)/33.5]^1.0^, *Q*M/*F* (L/h) = 425 × [BW (kg)/33.5]^0.75^, *V*_p_M/*F* (L) = 13,150 × [BW (kg)/33.5]^1.0^, and FM = 1.0 × {1 − 0.0654 × [6 − age (years)]} (if age is <6 years).

### Population pharmacokinetic analysis of DM-6705.

In order to model the metabolite DM-6705 along with delamanid, a joint model of delamanid and DM-6705 was simultaneously fitted to the data. The initial model of DM-6705 included a 2-compartment model with first-order metabolite formation (elimination of delamanid) and linear elimination. The mean fraction of delamanid metabolized to DM-6705 (fraction metabolized [FM]) was fixed to 1.0 (estimation would render the model structurally unidentifiable). Interindividual variability was included on the clearance of DM-6705 (CLM) and FM. After the assessment of the structural model, no further modification was done. Covariate assessment revealed a significant age effect (<6 years) on FM. The final parameter estimates are summarized in Table 2. Figure 2 presents GOF plots for the final population PK model. The pcVPCs are shown in Fig. 3.

### Data included in the concentration-QTc analysis.

The final PK/QT data set contained 354 valid QT measurements with time-matched observed delamanid/DM-6705 plasma concentrations from 37 participants. Also, corresponding time-matched baseline QT measurements were obtained on day −1. The distributions of samples over time after the last dose and delamanid/DM-6705 concentrations are presented in Fig. S4.

### Concentration-QTc analysis.

The relationship between heart rate (HR) and QT/QTc intervals (QT, QT corrected by Bazett’s formula [QTcB], and QT corrected by Fridericia’s formula [QTcF]) was evaluated via scatterplots using data at the drug-free state on day −1 (Fig. S5). The correlation coefficient (*R*sq) for QTcB (*R*sq = 0.044) was lower than that for QTcF (*R*sq = 0.484). Thus, QTcB was determined to be the preferred correction method for heart rate. No effects of delamanid/DM-6705 on HR or the time delay between delamanid/DM-6705 concentrations and ΔQTcB were graphically detected (Fig. S6). The assumption of a linear relationship between ΔQTcB and delamanid/DM-6705 concentrations was evaluated by plotting ΔQTcB against delamanid/DM-6705 concentrations (Fig. S7). The results suggest no major deviation from the linear assumption.

**FIG 4:**
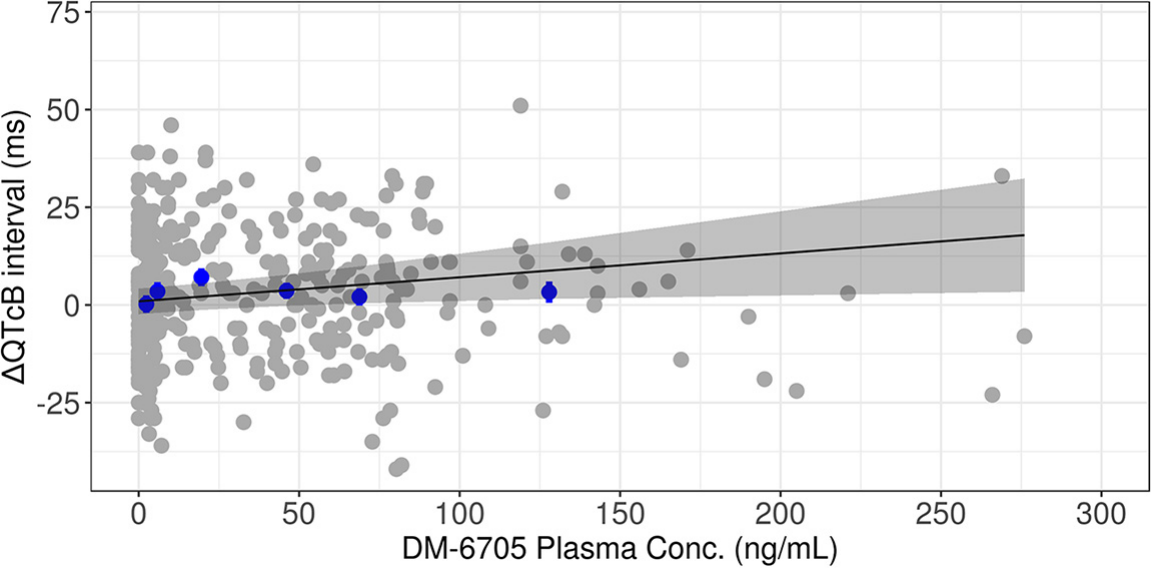
Observed and model-predicted ΔQTcB versus observed DM-6705 concentrations. Gray closed circles represent observed values. Blue closed circles and vertical bars represent binned observed data. The solid black line and gray-shaded area represent the model fit and 90% CI.

A linear mixed-effects model was applied to characterize the concentration-QTc relationship of delamanid/DM-6705. The estimate of the slope for the delamanid concentration was 0.00792 ms/(ng/mL) (90% confidence interval [CI], −0.00132, 0.0172) (Table S4). As the 90% CI of the slope included zero, the delamanid concentration was not determined to be a significant predictor of ΔQTcB. The estimate of the slope for DM-6705 was 0.0613 ms/(ng/mL) (90% CI, 0.016 to 0.107) (Table S5). Based on the CI of the slope, a significant impact of DM-6705 was detected on ΔQTcB. Observed and model-estimated ΔQTcB values versus observed DM-6705 concentrations are visualized in Fig. 4. A slight overprediction was observed, especially at higher exposure ranges.

### Simulated doses for matching adult PK.

The final population PK model was used to simulate and compare steady-state exposures in pediatric participants in different age/body weight groups. Demographic data were resampled from actual pediatric TB data, and 1,000 virtual participants were generated for each age/body weight group. The predicted exposure in pediatric participants was compared to the exposure in adults, using the approved delamanid dosage regimen for MDR-TB (100 mg BID). Based on these simulations, doses for pediatric participants within different age/weight groups that result in delamanid exposure (area under the concentration-time curve [AUC] at steady state) comparable to that in adults following the approved adult dose are summarized in Table 3. In addition, the model predictions of ΔQTcB at the maximum concentration (*C*_max_) of DM-6705 in pediatric participants following the maximum simulated dose were calculated using a linear mixed-effects model. The model-predicted upper bounds of the 90% CIs of the ΔQTcB values were <10 ms at the simulated *C*_max_ of DM-6705 following the predicted dose.

**TABLE 3:** Summary of calculated doses resulting in comparable adult exposures with the approved dose and model-predicted QTc prolongation with the maximum calculated dose^*c*^

Dosing interval, formulation, and age group	wt group (kg)	Calculated dose(s) comparable to adult exposure (mg)	Median AUC_0–24,ss_ (ng · h/mL)	AUC ratio vs adult	Simulated median *C*_max,ss_ of DM-6705 at highest dose (ng/mL)	Model-predicted ΔQTcB^*a*^ (ms) (90% CI)
QD dosing						
Film-coated tablet						
≤2 yrs	10–20	35–40	6,620–7,570	0.883–1.01	63.4	3.89 (1.17–6.60)
>2 yrs	10–20	35–50	6,160–8,800	0.821–1.17	87.8	5.38 (1.62–9.14)
	20–30	100	7,320	0.976	82.3	5.05 (1.52–8.57)
	30–40	Not achievable up to 100 mg				
	40–50					
	≥50					
Dispersible tablet						
≤2 yrs	10–20	40–50	6,370–7,970	0.849–1.06	66.0	4.05 (1.22–6.87)
>2 yrs	10–20	50	7,410	0.988	73.0	4.48 (1.35–7.60)
	20–30	100	6,160	0.821	68.6	4.21 (1.27–7.14)
	30–40	Not achievable up to 100 mg				
	40–50					
	≥50					

BID dosing						
Film-coated tablet						
≤2 yrs	10–20	20	7,570	1.01	61.5	3.77 (1.14–6.41)
>2 yrs	10–20	20–25	7,040–8,800	0.939–1.17	84.9	5.21 (1.57–8.84)
	20–30	30, 75 (50 mg a.m. + 25 mg p.m.)^*b*^	6,940, 8,670	0.925, 1.16	95.3	5.84 (1.76–9.92)
	30–40	35–50	6,300–8,830	0.840–1.18	95.4	5.85 (1.76–9.94)
	40–50	50	7,440	0.992	78.3	4.80 (1.45–8.15)
	≥50	50–100	6,240–7,900	0.832–1.05	81.8	5.02 (1.51–8.52)
Dispersible tablet						
≤2 yrs	10–20	20–25	6,370–7,970	0.849–1.06	64.4	3.95 (1.19–6.71)
>2 yrs	10–20	25–30	7,410–8,890	0.988–1.19	85.3	5.23 (1.58–8.88)
	20–30	35–40, 75 (50 mg a.m. + 25 mg p.m.)^*b*^	6,810–7,780, 7,300	0.908–1.04, 0.973	84.3	5.17 (1.56–8.78)
	30–40	40–50	6,060–7,580	0.808–1.01	81.2	4.98 (1.50–8.46)
	40–50	50–100	6,260–7,920	0.835–1.06	83.1	5.10 (1.54–8.65)
	≥50	100	6,650	0.887	68.5	4.20 (1.27–7.13)

a ΔQTcB is simulated by using the developed linear regression model and the simulated *C*_max,ss_ of DM-6705 after administration of the maximum dose, which provides exposure comparable to that in adults with the approved dose.

b Considering the availability of the tablet formulation, an additional dosing scenario, 50 mg (morning dose) plus 25 mg (evening dose), was simulated.

c QD, once a day; BID, twice a day. Given the limited number of participants in the <10-kg weight category (*n* = 5) in the analysis data set, dose calculations for participants weighing <10 kg are not provided.

## DISCUSSION

The disposition of delamanid and DM-6705 in pediatric participants with MDR-TB was described by a two-compartment model with linear elimination. This is consistent with the results from the adult population PK analysis (11). Since pharmacokinetic profiles of delamanid were highly variable even in the same subject, IOV was introduced on MAT and *F*_1_ to describe this variability.

Covariate analysis revealed a significant age effect on the bioavailability of delamanid, and a linear increase was observed from the ages of 0 to 2 years but not beyond. Since delamanid absorption is sensitive to meals, especially fat content, one possible explanation for this age-dependent increase is the difference in the quantity of food and fat content of meals consumed by infants compared with those consumed by older children. Additionally, a significant age effect was detected on FM (i.e., fraction metabolized from delamanid to DM-6705); the model suggests a linear increase in the FM up to 6 years old. This metabolism is mainly mediated by albumin (12), of which the plasma levels are reported to show modest increases by age during infancy and early childhood (up to 3 years old) (13). Interestingly, it has been reported that the unbound fraction of diazepam, which mainly binds to albumin, was 2-fold higher in 4-week- to 1-year-old children than in 1- to 3-year-old children, even though the increase in the mean albumin concentration was modest between these age groups (2.9 g/dL to 3.1 g/dL). This might be attributable to a lower affinity of albumin for the substrate as well as lower albumin levels in younger pediatric participants (14). Thus, both the qualitative and quantitative maturation of albumin might have an impact on the FM, and this may explain why age (and not the albumin amount) is the best predictor in the current analysis.

In addition to age, body weight, formulation, and dose were significant covariates. Due to its poor solubility, delamanid has shown a dose-dependent decrease in bioavailability in healthy adults and adults with MDR-TB. The same trend was observed in pediatric participants. In the full model after stepwise covariate modeling (SCM), additional covariates were detected as statistically significant covariates (such as BMI and total protein). However, the significance of these covariates is questionable since SCM has been reported to show selection bias with small numbers of participants (*n* < 50) (15) and limited power to detect true covariate-parameter relations as the model complexity increases (16). Also, highly correlated covariates were frequently selected in place of or in addition to the true covariates. Thus, correlated covariates (i.e., BMI with BW and total protein with albumin) were removed from the model. In addition, the significance of the covariate effect of albumin and sex is questionable, and these were removed from the model since the inclusion of these covariates was driven by very few individuals (see Fig. S1 in the supplemental material).

Steady-state delamanid exposures in pediatric participants predicted using the final population PK model and the dose that results in exposure comparable to that in adults following the approved adult dose were calculated. It should be noted that the calculated dose ranges are simply based on the exposure comparison of AUC values at steady state, and actual dose recommendations should be done considering other practical aspects such as the availability of tablet strengths (i.e., 50-mg film-coated and 25-mg dispersible tablets) or the convenience of patients/clinicians.

The efficacy of delamanid has been demonstrated thus far with a BID regimen in adults. Of note, a recent PK/pharmacodynamic (PD) analysis that utilized data from nonclinical, clinical, and hollow-fiber systems indicated that the AUC/MIC ratio is the major PK/PD driver for the efficacy of delamanid (17). Thus, a once-daily (QD) regimen that produces a delamanid AUC comparable to that in adults may provide efficacy similar to that of a BID regimen. Given the limited number of participants with a weight of <10 kg (*n* = 5) in the analysis data set and the limitation of the model and corresponding simulations, simulation for participants weighing <10 kg was not conducted.

Regarding the concentration-QT analysis, QTcB was determined to be a more appropriate correction for QTc than QTcF based on the slope of the regression line in this case (QTcB versus RR, *P* = 0.026 and *R*sq = 0.044; QTcF versus RR, *P < *0.01 and *R*sq = 0.484). This is in line with a previous report that evaluated various heart rate correction formulas in children (18). The investigation concluded that Bazett’s correction is more appropriate for trials involving infants and young children, in whom average heart rates tend to be well above 100 bpm. Also, S. Luo et al. reported correlation coefficients between HR and QT/QTc values for 4 different formulas (Bazett’s, Fridericia’s, Hodge’s, and Framingham formulas) for electrocardiogram (ECG) data from 10,303 normal ECG records (19). They showed that the performances of the formulas are different as the heart rate varies. For example, Fridericia’s correction has the least correlation with HR up to 99 bpm, whereas correlations between QTcF and HR are clearly higher than that for QTcB when the HR is over 100 bpm. The mean heart rate at baseline of our population was approximately 100 bpm; thus, it is not surprising that QTcB performed better in our data set.

For DM-6705, a significant positive correlation with QTcB was detected by linear mixed-effects modeling. In general, the cellular mechanism of a drug-induced prolonged QT interval is considered to involve the inhibition of the rapid component of the delayed rectifier potassium current (IKr) (20). Blocking of IKr leads to a prolongation of the ventricular action potential duration, leading to excess sodium influx or decreased potassium efflux. This excess of positively charged ions leads to an extended repolarization phase, resulting in a prolonged QT. Inhibition of the human ether-a-go-go-related gene (hERG) channel current, which conducts the rapid component of IKr, was investigated in studies using HEK-293 or CHO-K1 cells stably expressing the hERG channel. Inhibition was observed with delamanid and its metabolites DM-6704, DM-6705, and (4*RS*,5*S*)-DM-6720, and the most pronounced effects on hERG channel inhibition were observed with the predominant metabolite DM-6705 (50% inhibitory concentration [IC_50_] of 0.04 μg/mL) (7). Given the time course and the exposures of the metabolites in humans, it is likely that DM-6705 is a significant contributor to the observed QT prolongation.

The point estimate of the slope for DM-6705 [0.0613 ms/(ng/mL)] is consistent with the value in adults [0.0795 ms/(ng/mL)] (Otsuka internal report). The model showed a slight overprediction of ΔQTcB at higher exposure ranges (Fig. 4), which may lead to overprediction of the risk of QT prolongation at exposures of >75 ng/mL. This overprediction may be due to a slightly higher ΔQTcB at a lower concentration range (∼25 ng/mL). However, the linear model is well accepted for PK/QTc modeling by regulatory agencies (21) and, even with the overprediction, the increased QTc is unlikely to be clinically meaningful (see below).

Due to the lack of placebo data, ΔΔQTcB cannot be derived in this analysis. Thus, a small effect on the QT interval, i.e., a mean effect on the QTc interval that is not greater than approximately 5 ms, cannot be ruled out. However, the model-predicted upper bounds of the 90% CIs of the ΔQTcB value were all <10 ms at the simulated *C*_max_ of DM-6705 following the predicted dose, suggesting that there is unlikely to be a clinically meaningful effect on the QT interval. In addition to the lack of placebo data, there are some limitations in the current concentration-QT analysis. First, none of the participants in the analysis were on monotherapy with delamanid; all participants received an OBR consisting of several drug combinations, which could have had an impact on the QT interval. For example, fluoroquinolones (for example, levofloxacin) and clofazimine, which are reported to prolong the QT interval (22, 23), were used as part of the OBR. Macrolides (for example, clarithromycin), which are also reported to prolong QT (22), were used for a few patients as concomitant medications. Therefore, a confounding effect of these medications could have an effect on the QTc in those subjects. Second, the exposure range tested was limited, and the QT interval associated with supratherapeutic exposure was not tested.

QTc prolongation can be additive or synergic with other agents. The WHO recommends that delamanid may be added to the WHO-recommended longer regimen in children and adolescents with multidrug- or rifampicin-resistant TB (8). This treatment regimen is composed of at least pyrazinamide and four core second-line drugs considered to be effective. These include fluoroquinolones (levofloxacin/moxifloxacin), bedaquiline, linezolid, clofazimine, and cycloserine/terizidone. Among them, levofloxacin (22), moxifloxacin (24), bedaquiline (25), and clofazimine (23) are reported to prolong the QTc interval. In adult MDR-TB patients, although QTcF prolongation was detected in both the phase 2 and 3 trials with delamanid plus the OBR (levofloxacin and/or clofazimine), there appeared to be no clinically meaningful cardiac manifestations associated with this finding (26). Also, a phase 2 study was conducted to characterize the effect of coadministered bedaquiline and delamanid on the QTc interval (27). The study concluded that the QTc effects of bedaquiline and delamanid are not more than additive and were not associated with grade 3 or 4 QTc prolongation. Nevertheless, the clinical impact of QTc prolongation on pediatric patients receiving a broader range of background regimens should be further assessed.

In summary, a population PK model of delamanid and its major metabolite DM-6705 was developed in pediatric participants with MDR-TB. Covariate analysis revealed significant impacts of body weight, age, and dose on the delamanid PK. Dosages that provide exposure comparable to that in adults with the approved regimen have been calculated based on simulations from the population PK model. Based on linear mixed-effects modeling, delamanid concentrations did not have an impact on ΔQTcB, while DM-6705 concentrations had a modest impact. However, the model-predicted upper bounds of the 90% CIs of the ΔQTcB value were all <10 ms at the simulated *C*_max_ of DM-6705 with the maximum simulated doses, indicating the likelihood of a lack of a clinically meaningful effect.

## MATERIALS AND METHODS

### Trial design and subject population.

Data were obtained from two pediatric trials sponsored by Otsuka Pharmaceutical Development & Commercialization, Inc. Trial 232 was a phase 1, multicenter, open-label, uncontrolled, multiple-dose, age deescalation trial to assess the safety and tolerability of delamanid in pediatric participants with MDR-TB who were also receiving an OBR. This trial was conducted in 2 countries, Philippines and South Africa, and designed to define the pediatric dose in participants between 0 and 17 years of age that will result in delamanid systemic exposure equivalent to that observed in the pivotal adult trials where efficacy against MDR-TB has been demonstrated. Trial 233 was a phase 2, open-label, multiple-dose, multicenter trial to assess the safety, tolerability, PK, and efficacy of delamanid in pediatric participants with MDR-TB over a 6-month treatment period. This long-term trial was an extension of trial 232 and conducted in participants in Philippines and South Africa who had completed trial 232. The goals of this extension trial were to evaluate the long-term safety and tolerability of the age-specific delamanid doses used in trial 232 and to determine the dose in pediatric MDR-TB participants that will result in delamanid plasma exposure similar to the effective plasma exposure in adult MDR-TB participants.

Both trial 232 and trial 233 were age deescalation trials in which pediatric participants were enrolled in 4 age groups: adolescents aged 12 to 17 years, inclusive (group 1); children aged 6 to 11 years, inclusive (group 2); children aged 3 to 5 years (group 3); and infants from birth to 2 years of age (group 4). Details on dosing regimens are described in Table S1 in the supplemental material.

In accordance with the International Council for Harmonisation good clinical practice consolidated guideline and applicable local laws and regulatory requirements of the countries in which the trial was conducted, copies of the protocol, any amendments, and the informed consent form were reviewed and approved by the governing institutional review board or independent ethics committee for each investigational site or country, as appropriate.

### Pharmacokinetic sampling strategies and assays.

Blood collection for PK occurred as shown in Table S1. Approximately 3 mL of blood was collected per PK sample for the participants in group 1, 2 mL was collected per PK sample for participants in groups 2 and 3, and 0.6 mL was collected per PK sample for participants in group 4. Determination of the total (i.e., free plus protein-binding) concentrations of delamanid and DM-6705 in human plasma samples was conducted by the ultraperformance liquid chromatography-tandem mass spectrometry (UPLC-MS/MS) method, which had adequate linearity, specificity, precision, and accuracy. The lower limit of quantitation and the upper limit of quantitation were 1.00 and 500 ng/mL, respectively, for both delamanid and DM-6705.

### ECG measurements.

Twelve-lead ECGs were recorded with the subject in a supine or semirecumbent position and at rest for ≥10 min (as possible for groups 3 and 4). ECGs were performed before PK blood draws when assessments were scheduled at the same time (Table S1). Three tracings were obtained at each time point (approximately 3 to 5 min between tracings), with the ECG leads left in place. Heart rate, PR interval, QRS interval, QT interval, and QT interval corrected by Fridericia’s and Bazett’s formulas (QTcF and QTcB, respectively) were recorded. All ECGs were analyzed by a specialized central laboratory.

### Population pharmacokinetic modeling.

The population PK of delamanid and DM-6705 was described with nonlinear mixed-effects models using NONMEM software (Icon Development Solutions, Hanover, MD, USA). First-order conditional estimation with η-ε interaction was used for the modeling. The population PK of delamanid and DM-6705 was sequentially modeled: the population PK model of delamanid was developed first, the concentration data of DM-6705 were then added, and a joint model was simultaneously fitted to all data. Concentrations of both delamanid and DM-6705 were converted into molar units for modeling. All measurements reported as being below the lower limit of quantitation were considered missing in the analysis data set and ignored during the analysis.

For structural model development, combinations of the scenarios shown in Table S2 were tested. The number of transit compartments was determined by the stepwise addition of transit compartments to minimize the objective function value (OFV). Covariate assessment was performed by the SCM approach (28). The significance levels for the forward-addition and backward-deletion steps were 0.01 and 0.001, respectively. Table S3 summarizes the covariates evaluated and the PK parameters on which each was tested.

Unless otherwise specified, continuous and categorical covariates were incorporated into the population model as follows:

$$\text{Continuous covariate}:\;P_{j}={\text{θ}}_{k}\cdot {\left(\frac{X_{\mathrm{ij}}}{X_{j\text{,med}}}\right)}^{\;{\text{θ}}_{i}}$$

$$\text{Categorical covariate}:\;P_{j}={\text{θ}}_{k}\cdot {\left(1\;+{\text{θ}}_{i}\right)}^{X_{\mathrm{ij}}}$$

where *P_j_* is the *j*th population estimate of parameters, *X_ij_* is the covariate of subject *i* for the parameter *P_j_*, *X_j_*_,med_ is the median of covariate *X* for the subject population, θ*_k_* is the typical value of the parameter *P_j_*, and θ*_i_* is a coefficient that reflects the covariate’s effect on the parameter.

The interindividual/occasional random effects on the parameters were modeled assuming the multiplicative form given by the expression θ*_ki_* = θ*_k_* · $e^{\eta_{\mathrm{ki}}}$, where θ*_k_* denotes the typical parameter value and η*_ki_* denotes the interindividual/occasional random effect for the *i*th subject/*i*th dosing occasion, assumed to have a mean of zero and variance ω^2^.

The residual-error structure was assumed to follow a proportional-error model described by *Y_ij_* = *C_ij_* · (1 + ε_1_*_ij_*), where *Y_ij_* is the *j*th observed concentration for the *i*th subject, *C_ij_* is the corresponding predicted concentration, and ε*_ij_* is the residual error under the assumption that ε ∼ *N*(0,σ^2^).

### Concentration-QT analysis.

The relationship between the QTc and the plasma concentration of delamanid and DM-6705 was examined. The time-matched change from the baseline QT was computed for each subject as follows: (i) for each subject at each time point, the triplicate QTc values were averaged, and (ii) the change from the baseline for each time point (ΔQTc) was computed as the value at the time point minus the value at baseline. Before conducting regression analysis, the following were graphically evaluated: (i) the heart rate (HR) correction for QTc was assessed via scatterplots of QT/QTc and RR intervals using data at baseline; (ii) the potential effect of the drug on HR was evaluated by plotting the time course of the mean change from the baseline in HR (ΔHR) effects by treatment; (iii) the potential delayed effect of the drug concentration on ΔQTc was evaluated by assessing the mean concentration and ΔQTc profiles by treatment and time in a hysteresis plot; and (iv) the assumption of a linear concentration-QTc relationship was assessed by a concentration-ΔQTc plot incorporating trend lines (i.e., locally estimated scatterplot smoothing [LOESS] smooth and linear regression lines).

A linear mixed-effects model was used to examine the relationship between the time-matched baseline-corrected QTc and the plasma concentration. The model used ΔQTc as the dependent variable with plasma concentration and time as fixed effects and subject as a random effect as ΔQTc*_ijk_* = (θ_0_ + η_0,_*_i_*) + (θ_1_ + η_1,_*_i_*) · *C_ijk_* + θ_2_ · (QTc*_ij_*_0_ − $\bar{{\text{QTc}}_{0}}$), where ΔQTc*_ijk_* is the time-matched baseline-corrected QTc for subject *i* in treatment *j* at time *k*; θ_0_ is the population mean intercept in the absence of a treatment effect; η_0,_*_i_* is the random effect associated with the intercept term θ_0_; θ_1_ is the population mean slope of the assumed linear association between the concentration of delamanid and QTc*_ijk_*; η_1,_*_i_* is the random effect associated with the slope θ_1_; *C_ijk_* is the concentration of delamanid for subject *i*, treatment *j*, and time *k*; θ_2_ is the fixed effect associated with the baseline; and $\bar{{\text{QTc}}_{0}}$ is overall mean QTc*_ij_*_0_, i.e., the mean of all the baseline (time zero) QTc values. All random effects on the parameters were modeled assuming a normal distribution. The confidence intervals of fixed effects are calculated based on the standard errors of parameter estimates. The graphical and linear mixed-effect modeling analyses were performed using R version 3.5.1 (R Project [http://www.r-project.org/]).

### Simulation to calculate matching dose and QTc prolongation.

The final population PK model was used to simulate steady-state exposure in pediatric participants in different age/body weight groups. Demographic information (i.e., age and body weight) was extracted from two studies in pediatric participants with TB (*n* = 266). Study 1 is an observational study in pediatric participants with MDR-TB, while study 2 is a hospital-based study for pediatric participants who started TB treatment (not MDR). Both studies were conducted in South Africa. The observed median AUC from 0 to 24 h at steady state (AUC_0–24,ss_) of delamanid in adults with MDR-TB administered 100 mg BID for 2 months (trial 204), i.e., 7,500 ng · h/mL, was chosen as the target for the simulations. Doses that produced exposures similar to this target AUC (i.e., the median ratio of the AUC_0–24_ at steady state within ∼0.80 to 1.25 to the target) were defined as doses sufficiently similar to those for adults. Also, the model-predicted ΔQTcB at steady-state *C*_max_ (*C*_max,ss_) with the maximum predicted dose was calculated using simulated exposures and linear mixed-effects models for QTc.
