# Supplementary material for: Population Pharmacokinetic and Concentration-QTc Analysis of Delamanid in Pediatric Participants with Multidrug-Resistant Tuberculosis
Source: Antimicrob Agents Chemother. 2022 Feb 15;66(2):e01608-21. doi: 10.1128/AAC.01608-21 (PMC8846319; doi:10.1128/AAC.01608-21)
Supplement: Supplemental file 1 — Supplemental tables and figures. Download AAC.01608-21-s0001.pdf, PDF file, 3.4 MB [file aac.01608-21-s0001.pdf]

Supplementary Information for

Population Pharmacokinetic and Concentration-QTc Analysis of Delamanid in Pediatric Participants with Multidrug-Resistant Tuberculosis

Supplemental Tables

Table S1. Dosing Regimens and Pharmacokinetic/Pharmacodynamic Sampling Plans

Trial Number/ Phase	Dosing Regimen	Pharmacokinetic Sampling	Pharmacodynamic (QTc) Sampling
232/<br>Phase 1	Delamanid 50 mg oral tablet or 25 mg/5 mg dispersible tablet[c] for 10 days<br>• Group 1 (ages 12 - 17 years):<br>100 mg BID + OBR, adult formulation tablet<br>• Group 2 (ages 6 - 11 years):<br>50 mg BID + OBR, adult formulation tablet<br>• Group 3 (ages 3 - 5 years):<br>25 mg BID + OBR, dispersible tablet<br>• Group 4 (ages birth - 2 years):<br>dispersible tablet<br>➢ 10 kg: 10 mg BID + OBR<br>➢ 8 kg and ≤ 10 kg: 5 mg BID + OBR<br>➢ ≥ 5.5 kg and ≤ 8 kg: 5 mg QD + OBR	• Days 1 to 2 and 10 to 11:<br>➢ Predose and 2, 4, 10, 12[a], 14, and 24 hours postdose<br>• Days 13[a], 15, and 18 (72, 120, and 192 hours, respectively, postdose on Day 10)<br>• Early Termination	• Day -1: Time of day that corresponds to the predose, 4-hour, and 10-hour post morning dose administration on Day 1<br>• Days 1 and 10: Predose, 4-hour, and 10-hour post morning dose administration<br>• Day 18: 192-hour post dose<br>• Early Termination: Time paired with PK blood sample
233/<br>Phase 2	Delamanid 50 mg oral tablet or 25 mg/5 mg dispersible tablet[c] for 6 months<br><br>Dosing regimen is same as 242-12-232	• Predose on Days 1, 56, 154, and 182<br>• Theoretical predose on Day 210[b] (Sampling timing is same as other predose samples, but no delamanid dose is planned)<br>• Any time point on Days 14, 98, 189, 196, 203, and 238[b]<br>• Early Termination	• Day -1<br>• Predose on Days 28, 84, and 126<br>• Days 1, 56, 154, and 182: Predose, time paired with PK blood sample<br>• Day 210: Theoretical predose, time paired with PK blood sample<br>• Early Termination

[a] The samples at 12 hours postdose and Day 13 are only available for Groups 1 and 2. [b] Last dose of delamanid on Day 182.
[c] 25 mg and 5 mg dispersible tablet are delamanid pediatric formulations. QD=Once a day. BID=Twice a day.

Table S2. Combination of Scenario Tested in Structural Model Development

Compartmental Model	Absorption Order	Lag Time for Absorption	Residual Error Model
One<br>Two<br>Three	Transit compartment<br>First-order	Yes<br>No	Additive<br>Proportional<br>Additive and Proportional

Table S3. Planned Evaluation of Covariate in Analyses

**Covariate**	**Pharmacokinetic Parameters**		
	Absorption Rate/Extent	Volume of Distribution	Clearance
Dose	X		
Formulation	X		
Dose timing	X		
Age Group number	X		
Age	X	X	X
Body weight		X	X
Body surface area		X	X
BMI	X	X	X
Sex	X	X	X
Race	X	X	X
Country	X	X	X
Clinical laboratory values [a]	X[b]	X[b]	X

[a] Clinical laboratory values include estimated glomerular filtration rate using Schwartz equation, albumin, alanine aminotransferase, aspartate aminotransferase, total bilirubin, alkaline phosphatase, and total protein.

[b] only albumin was tested.

Table S4. Parameter Estimates of the Linear Mixed Effects Model for Delamanid/ΔQTcB

**Parameter**	**Estimate**	**SE**	**90% CI**
**Fixed Effects**			
θ_0, Intercept (ms)	1.47	1.67	-1.80, 4.75
θ_1, Slope (ms/[ng/mL])	0.00792	0.00471	-0.00132, 0.0172
θ_2, Baseline QTcB effect	0.0318	0.0739	-0.113, 0.177
**Inter-Individual Variability (shown as variance)**			
η_0, Random effect for the intercept	63.5	NE	
η_1, Random effect for the slope	0.0000937	NE	
**Residual Variability (shown as variance)**	180	NE	

NE: not estimated

Table S5. Parameter Estimates of the Linear Mixed Effects Model for DM-6705/ΔQTcB

Parameter	Estimate	SE	90% CI
**Fixed Effects**			
θ_0, Intercept (ms)	0.923	1.61	-2.22, 4.07
θ_1, Slope (ms/[ng/mL])	0.0613	0.0231	0.016, 0.107
θ_2, Baseline QTcB effect	0.0309	0.0728	-0.112, 0.174
**Inter-Individual Variability (shown as variance)**			
η_0, Random effect for the intercept	59.9	NE	
η_1, Random effect for the slope	0.00446	NE	
**Residual Variability (shown as variance)**	174	NE	

NE: not estimated

Supplemental Figures

Figure S1. Impact by Removing Individuals on Significance of Covariates (Upper: Albumin, Lower: Sex)

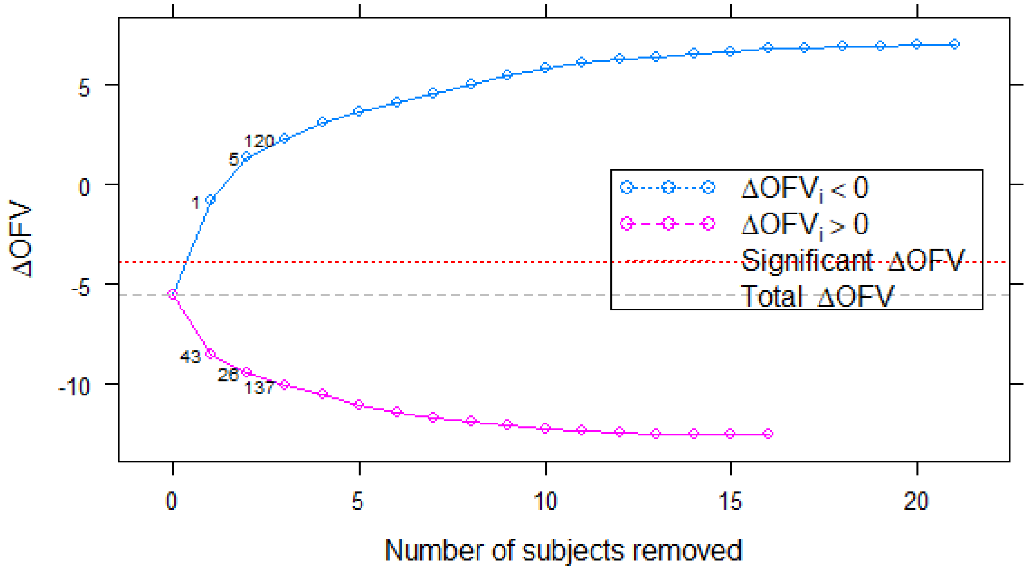

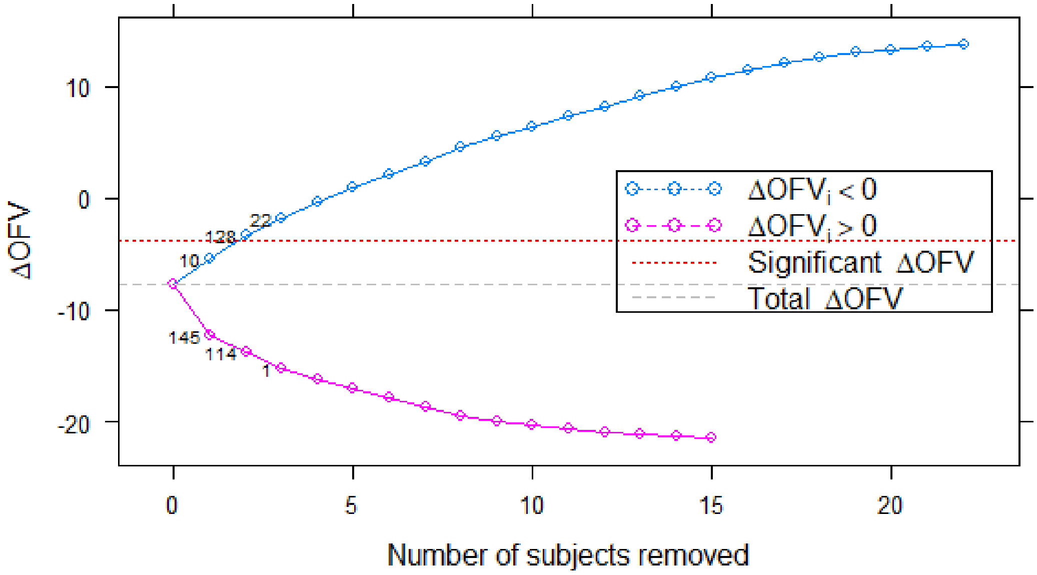

Each plot represents ΔOFV (OFV with covariate [albumin or sex] - OFV without covariate) by removing individuals. Blue or pink line represents runs with increased or decreased ΔOFV by removing subjects. Red line represents the significance threshold for ΔOFV with *P*<0.05 and degree of freedom = 1.

Gray line represents ΔOFV by using all data. Each number is ID of subject removed.

Figure S2. Goodness of Fit Plot of Final Model Stratified by Analyte and Age Group

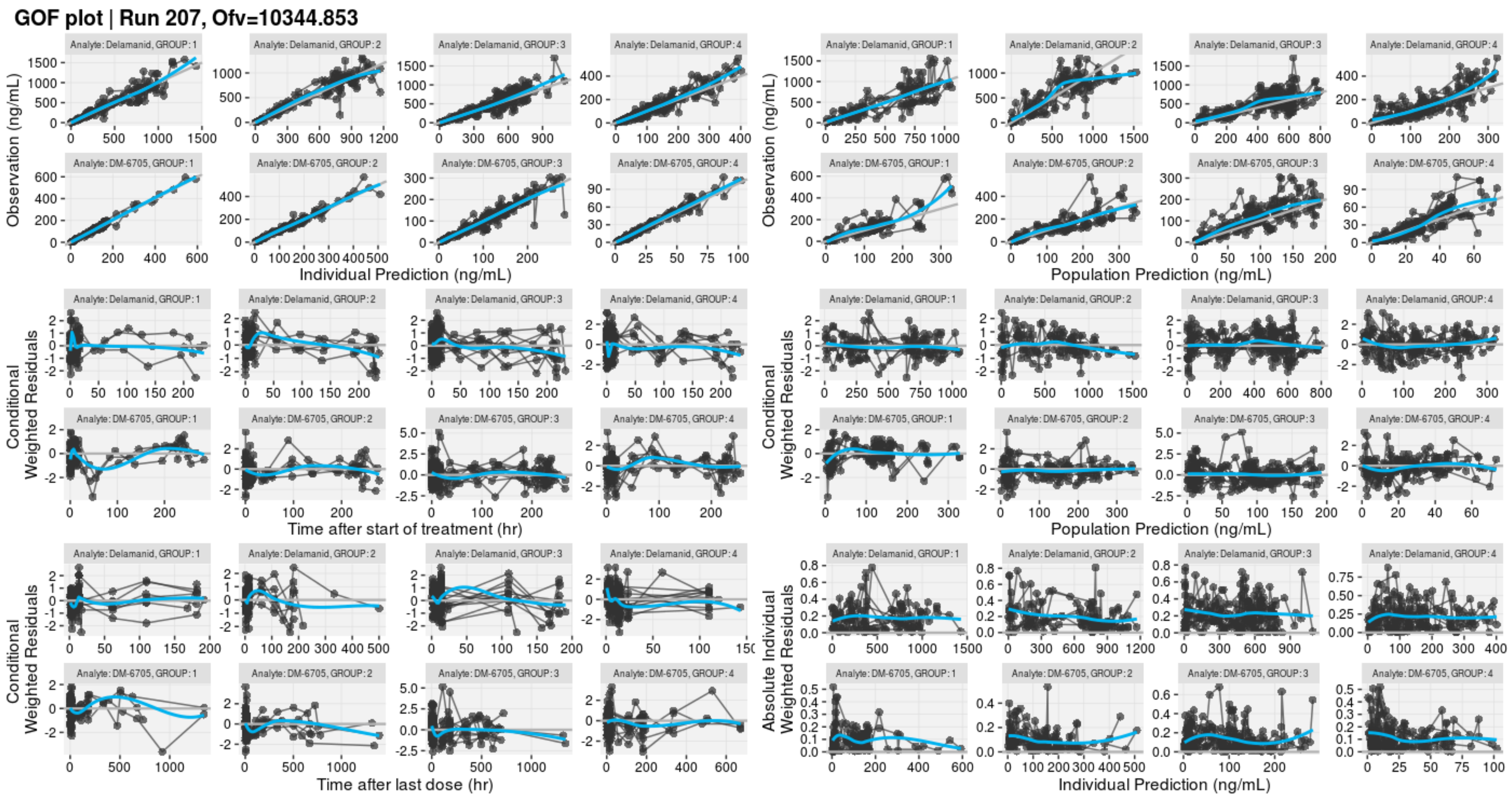

Black circle represents individual data. Connected line means data from same individual.
Gray line represents line of identity (y=x) or y=0. Blue line shows smoothing line of data.
Group 1: ages 12 - 17 years, Group 2: ages 6 - 11 years, Group 3: ages 3 - 5 years, Group 4: ages birth - 2 years.

Figure S3. Prediction-Corrected Visual Predictive Check Plots by Age Group and Analyte

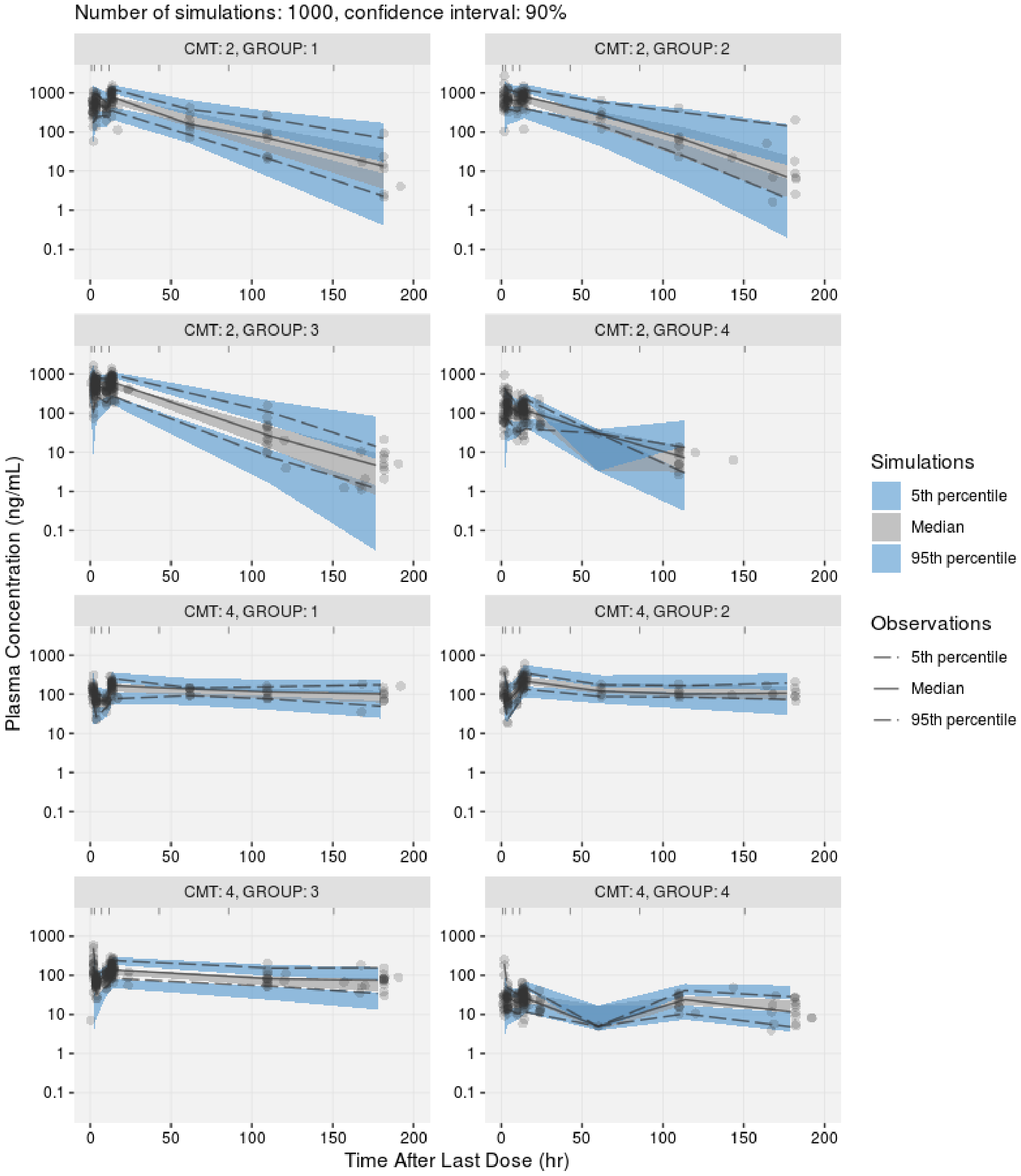

CMT: 2 represents delamanid and CMT: 4 represents DM-6705.
Group 1: ages 12 - 17 years, Group 2: ages 6 - 11 years, Group 3: ages 3 - 5 years, Group 4: ages birth - 2 years

Figure S4. Distributions of QT Samples Stratified by Study

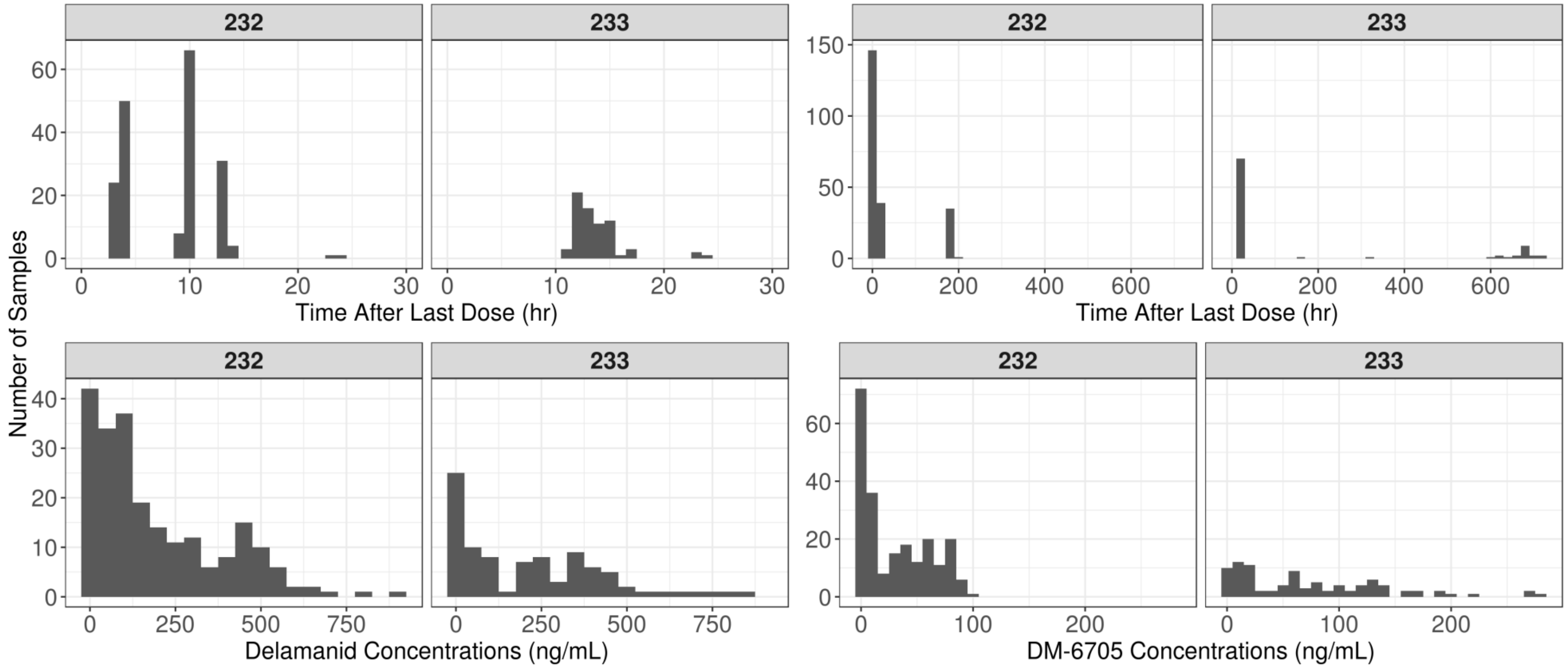

Left top figure and right top figure are basically same figures, but zoomed in 30 hr after dose for left top one.

Figure S5 QT/QTc versus RR Plots

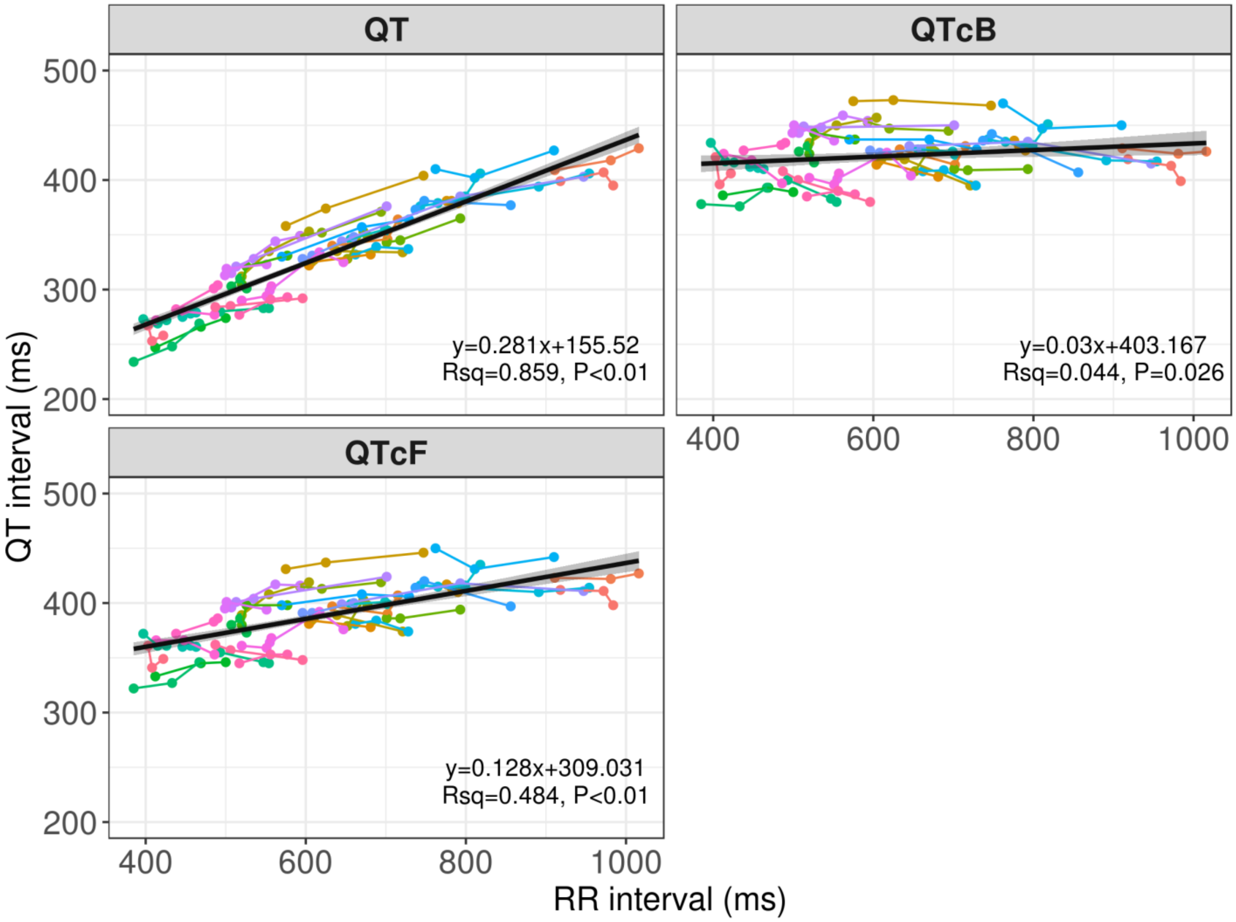

Closed circles represent observed data.
Each line represents data from one subject.
Black solid line with grey shaded area represent linear regression line with 90% CI.
Rsq: R squared.

Figure S6 Hysteresis Plot: Mean ΔQTcB versus Mean DM-6705 Concentration by Cmax Quantiles of Delamanid (Left)/DM-6705 (Right) Stratified by Study Day

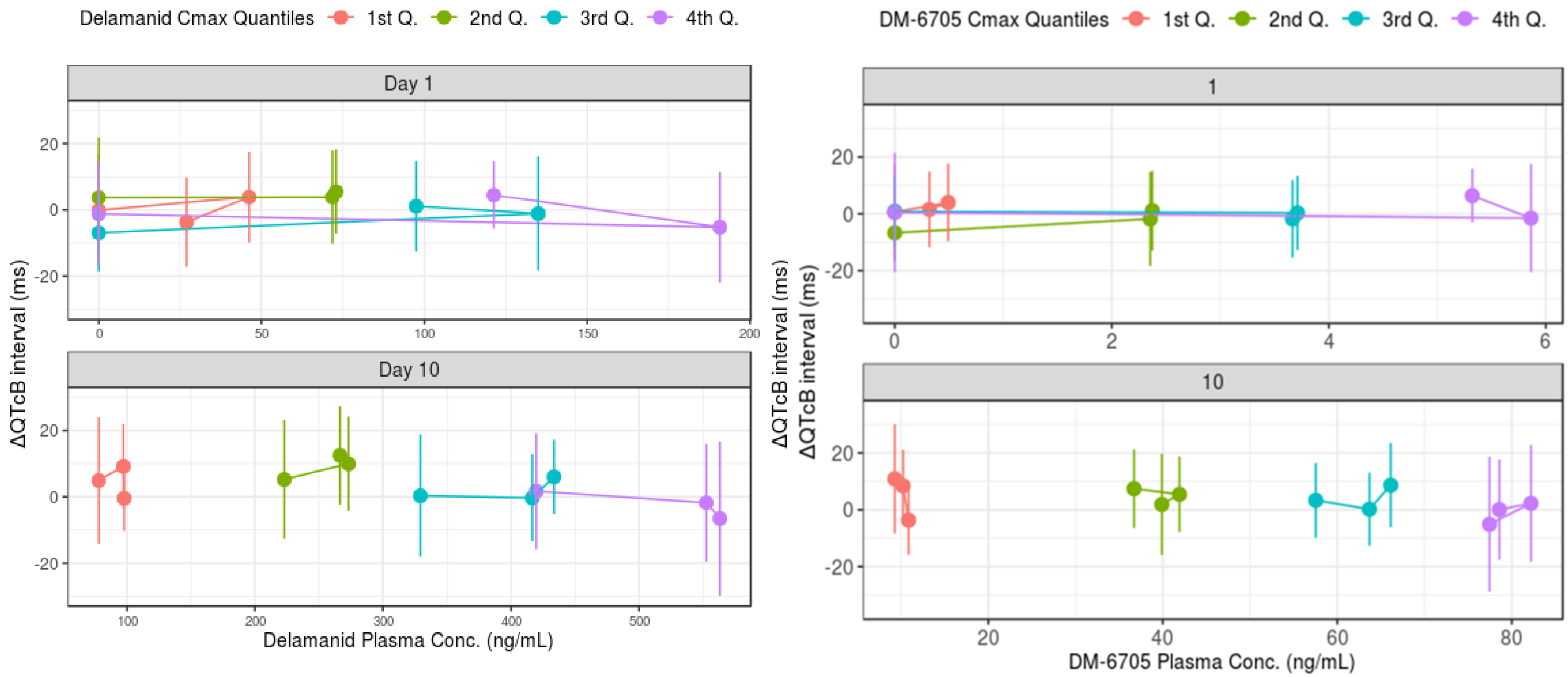

Data are shown as mean ± SD.

ΔQTcB is baseline-corrected QTcB.

Q: quantile.

Figure S7 Scatter Plot of ΔQTcB vs Concentration

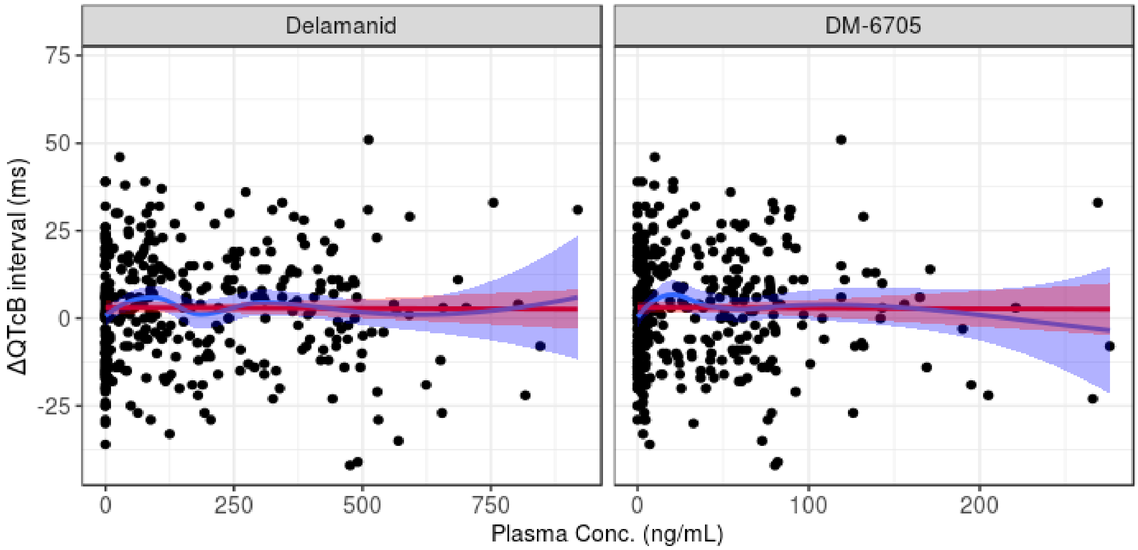

Closed circles represent observed values.
Red solid line and shaded area represent linear regression line and its 90% CI.
Blue solid line and shared area represent loess smoothing curve and its SE.
